# Artificial Intelligence in Nursing: Technological Benefits to Nurse’s Mental Health and Patient Care Quality

**DOI:** 10.3390/healthcare12242555

**Published:** 2024-12-18

**Authors:** Hamad Ghaleb Dailah, Mahdi Koriri, Alhussean Sabei, Turky Kriry, Mohammed Zakri

**Affiliations:** College of Nursing and Health Sciences, Jazan University, Jazan 45142, Saudi Arabia; mkurairy@jazanu.edu.sa (M.K.); amsabei@jazanu.edu.sa (A.S.); tkriry@jazanu.edu.sa (T.K.);

**Keywords:** artificial intelligence in nursing, patient care, digital health, nurse wellbeing

## Abstract

Nurses are frontline caregivers who handle heavy workloads and high-stakes activities. They face several mental health issues, including stress, burnout, anxiety, and depression. The welfare of nurses and the standard of patient treatment depends on resolving this problem. Artificial intelligence is revolutionising healthcare, and its integration provides many possibilities in addressing these concerns. This review examines literature published over the past 40 years, concentrating on AI integration in nursing for mental health support, improved patient care, and ethical issues. Using databases such as PubMed and Google Scholar, a thorough search was conducted with Boolean operators, narrowing results for relevance. Critically examined were publications on artificial intelligence applications in patient care ethics, mental health, and nursing and mental health. The literature examination revealed that, by automating repetitive chores and improving workload management, artificial intelligence (AI) can relieve mental health challenges faced by nurses and improve patient care. Practical implications highlight the requirement of using rigorous implementation strategies that address ethical issues, data privacy, and human-centred decision-making. All changes must direct the integration of artificial intelligence in nursing to guarantee its sustained and significant influence on healthcare.

## 1. Introduction

Artificial intelligence (AI) is the application of technologies that allow machines to carry out tasks that require human intelligence, including decision-making, learning, and reasoning. Its uses span sectors, including banking for fraud detection, transportation for driverless cars, and education for individualised learning. In healthcare, artificial intelligence greatly enhances the nature of operations, diagnostics, and clinical procedures. Specifically, in nursing, it helps with patient monitoring, recordkeeping, and scheduling using predictive analytics, virtual companions, and chatbots. Its integration in healthcare has rapidly increased in recent years and is revolutionising the delivery of medical services. Studies suggest that there is a highly fruitful and practical application of various aspects of AI, such as machine learning algorithms, natural language processing, and predictive analytics, to improve patient care, simplify administrative processes, and support clinical decision-making [1,2,3,4,5]. Artificial intelligence can also enhance human capacities, streamline operations, provide creative answers to current issues in patient care, and reduce healthcare costs. In the US alone, healthcare expenditure rose to 18% from 5% from 1960 to 2022, and the US administration considered it due to ineffective technologies in healthcare management [6]. At the same time, healthcare institutions that have accepted artificial intelligence have met their 25% operating cost reduction targets and improved patient outcomes [7]. In addition, AI is also known to assist in alleviating professional crises in healthcare providers, like stress, burnout, and compassion fatigue [8,9].

Nurses, regarded as primary healthcare professionals, are the backbone of patient care. Nurses are frontline caregivers with numerous responsibilities, including direct patient care, medication administration, the monitoring of vital signs, assisting various medical procedures, managing challenging clinical environments, and patient emotional support [10]. They serve as crucial links between the patient and physicians. Their communication skills and compassionate approach to patients and families are essential for positive health outcomes [11]. Their expertise and dedication thus contribute to the advancement of public health standards.

Although nurses have considerable obligations in the healthcare industry, their potential to provide high-quality treatment is usually hampered by strict shift patterns, degrees of experience, occupational dynamics, nurse-to-patient ratios, and heavy workloads. When combined with professional discontent and conflicts, these demands can develop into occupational stresses that aggravate burnout and influence the quality of patient treatment [12]. Technological additions, especially emerging technologies like AI, can substantially improve the skills and outcomes of nursing care. Unlike the applications of AI in other industries, such as banking or manufacturing, where the main emphasis is on efficiency and automation, AI in nursing is especially meant to complement the human-centred character of patient care. It seeks to reduce nurses’ workload so they may concentrate more on direct patient contact and ensure that technology improves rather than replaces the compassionate elements of providing care. Therefore, AI integration can enhance patient care efficiency, increase patient safety, and reduce the burden on nurses (Table 1). AI-driven tools can assist nurses in analysing large datasets, predicting potential complications, and thus improving decision-making and cognitive workload [13,14]. For example, AI can promptly alert a nurse ahead of time to the deteriorating conditions of a patient, and nurses can therefore provide early and effective intervention [15]. Additionally, AI tools can assist with patient education, symptom triage, and remote monitoring, thus freeing nurses to focus on more human-centred aspects of patient care [16].

This review aims to explore the transformative role of AI in enhancing mental support for nurses and look into opportunities for human–AI collaboration to reduce the workload, improve productivity, and provide personalised support for mental wellbeing. Furthermore, this review will analyse the ethical considerations of AI integration in nursing. This review aims to provide a balanced understanding of AI integration’s potential benefits and challenges by addressing these aspects, offering insights into future AI–human collaboration prospects in nursing.

## 2. Materials and Methods 

### Literature Search

This comprehensive review was conducted by searching databases like Google Scholar and PubMed for relevant papers, book chapters, and reliable web sources. Using the following keywords, the search concentrated on English-written works: artificial intelligence, nursing, mental health support, mental distress, burnout, patient care, digital health, stress, predictive analytics, machine learning, chatbots, robots, conversational AI, patient monitoring system, ethics, data privacy, safety, security, bias. The Boolean operators “AND” and “OR” were applied to refine the search and explore interrelated topics. The search results were evaluated, and then Zotero software imported data for reference management. Duplicate entries were eliminated before additional investigation. Articles were shortlisted for thorough reading depending on their relevance to the study’s main goals. Titles and abstracts were examined to identify studies addressing the integration of artificial intelligence in nursing, its use in mental health support, the enhancement of patient care, and related ethical questions. All of the authors equally participated in searching, sorting, and investigating the collected literature. The search was conducted over six months from 3 June 2024.

Inclusion Criteria

The reviewed articles comprised those that satisfied the following requirements:

1. Published in English.
2. Drawn from book chapters, theses, peer-reviewed publications, conference proceedings, and credible web sources.
3. Focusing on AI in mental health support, patient care, and related ethical issues.
4. Published within the past 40 years.

Exclusion Criteria

Articles were not included depending on the following standards:

1. Non-English works.
2. Research including dubious or lack of data.
3. Review articles or opinion pieces devoid of main conclusions.
4. Articles irrelevant to the theme, such as those concentrated only on artificial intelligence outside of the framework of nursing or healthcare.

This methodology guaranteed an extensive and targeted literature analysis, helping to identify pertinent studies to investigate artificial intelligence and nursing practice with respect to nurses’ mental health and uncompromised patient care.

## 3. The Mental Health Landscape of Nurses

**Figure 1:**
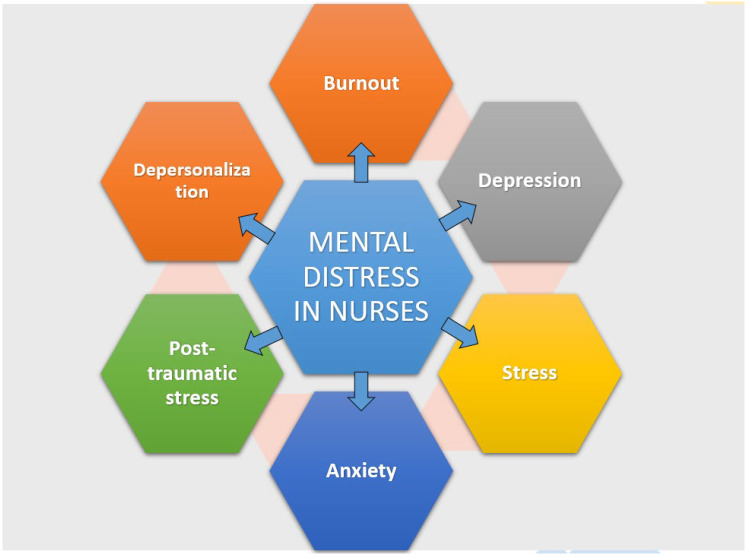
Mental distress experienced by nurses.

Nurses across the globe have played a remarkable role in healthcare. They handle patients in normal clinical settings and critical care units. Their heroic role during the COVID-19 outbreak is commendable, and many even lost their lives during their dedicated care delivery [26]. Numerous studies identify various occupational health hazards encountered by nurses [27,28]. Being frontline fighters, they are constantly exposed to patients’ pain, trauma, and loss of life. They must maintain presence of mind under these circumstances, sometimes while working in compromised work settings and work attire, such as during the COVID-19 pandemic [29]. They also work extended hours, often have inadequate personal protective equipment, and must keep themselves away from their family. Even before the COVID-19 pandemic, nurses were susceptible to high rates of mental distress. The mental stress afflicting nurses constitutes stress, post-traumatic stress, burnout, anxiety, depression, and even depersonalisation (Figure 1). All of these circumstances adversely affect the wellbeing of nurses and healthcare delivery. It is no surprise that nursing is considered a stressful career around the world [30]. A recent study has shown that nursing stress predicts the emotional wellbeing of neonatal intensive care unit nurses, highlighting its significant impact on mental health [31]. Some of the common distress-related conditions that develop in nurses are anxiety, depression, and post-traumatic stress disorder, for which their profession itself is the primary underlying reason. Studies also suggest that nurses are more prone to developing these mental health problems than doctors working in the same setting [32,33]. The long-term impacts of stress can be profound and wide-ranging, negatively influencing their job performance and quality of care. Further, under this distress, there is a chance of developing job dissatisfaction, eventually leading to nurses leaving the profession [34]. Studies from Ethiopia and Iran have shown that 48.8% and 91.2% of nurses, respectively, experienced stress because of their job [35,36].

Burnout, another problem demonstrating similar symptoms to stress, tends to develop gradually over time but, at later stages, is greater in intensity. Burnout could be a consequence of stress or vice versa [12]. Sleeplessness, prolonged stress, fatigue, helplessness, diminished concentration, exhaustion, etc., are common symptoms of burnout. A recent study estimated the prevalence of burnout in nurses at 50% [37]. Several reasons can trigger burnout in nurses, including the work environment. For instance, a study from southern Italian hospitals conducted with 169 healthcare professionals, 64.5% of whom were nurses, assessed the connection between perceived burnout levels and the work environment. The results showed that levels of stress and burnout were much raised by inadequate environmental comfort and safety conditions [38]. The long-lasting effects of burnout can affect job performance, and the consequences are not limited to the nursing practitioner themselves, but also to the work environment and the individuals associated with and/or interacting with them [12,39]. Thus, it is also a social concern besides being a concern to the individual themselves. As in the case of stress, burnout can lead to job dissatisfaction, eventually resulting in the quitting of the profession altogether [40].

The incidence of mental distress is not limited to stress, post-traumatic stress, and burnout. Studies have demonstrated that anxiety and depression are also prevalent. A recent study from India has shown that, among the occupational mental health conditions experienced by nurses, stress, anxiety, and depression accounted for 50.8%, 74%, and 70.8%, respectively, with at least 79.1% of the nurses having any one of them [41]. Studies from other countries indicate that these problems are widespread across the globe. For example, studies showed that 43.1% and 38.5% (Saudi Arabia), 18.1% and 34.3% (China), 50.3% and 51.9% (UK), 38.8% and 37.4% (Iran), and 39.1% and 6.3% (France) of nurses in various countries had anxiety and depression, respectively [42,43,44,45,46].

## 4. Impact of Mental Distress of Nurses on Patient Care

The impact of mental trauma is a critical issue in healthcare, as the wellbeing of nurses is linked to patient care. It is well known that there is a severe shortage of nurses in the healthcare industry [47,48,49,50]. This results in nurses becoming overburdened as there is a unique requirement to show empathy while taking care of the needs of the patients. However, nurses become physically and mentally exhausted, which affects their ability to deliver safe, quality care [51]. The consequences of mental distress for nurses include compromised patient safety and an increased number of errors [52,53]. For instance, a study by Baye and colleagues [54] reported that higher workloads and stress were related to an increased rate of medication errors, which seriously impacted patient safety. Another study in 2021 found that nurses with diminished physical and mental health made more medical errors than those with better health [55]. An increased rate of healthcare-associated infections is also related to poor patient outcomes [56,57] due to nurses’ mental distress. The consequences may even be life-threatening to patients [58]. Those nursing staff prone to making these mistakes typically endure one or more conditions like fatigue, impaired cognitive function, and lack of focus. Nurses may also progressively lose empathy or emotionally distance themselves from their patients, therefore seeing their patients as objects rather than as people who need caring treatment. One might describe such a state by means of the depersonalisation concept. Critical care nurses from a tertiary hospital in Malaysia exhibited a prevalence of depersonalisation as high as 72.9% [59]. As a result of depersonalisation, there are negative outcomes in terms of patient experience and the rapport between the nurse and the patient. The results of a 2021 study indicated that patients who were receiving care from nurses experiencing burnout had worse patient satisfaction [60]. A thorough study conducted in the same year revealed that patients treated by nurses with burnout (characterised by depersonalisation) reported lower satisfaction levels [61].

Absenteeism and staff turnover are two other consequences of mental health issues in nurses that negatively affect patient care and quality. Stress and burnout can lead to understaffing due to the inability of nurses to work under these conditions. As a result, the remaining nurses face increased workloads, resulting in a further decline in quality [61]. Higher turnover rates also mean that healthcare facilities are frequently forced to take on new nursing staff, which leads to a lack of continuity in care. The inverse relation between nurse turnover and health outcomes was documented in a study conducted in 2022 [62]. Nurse turnover also prolongs the stay of patients in the hospital and increases readmission rates as new staff tend to take time to adapt to the new work environment [63].

The growing concern about mental health, productivity, and quality of patient care calls for innovative solutions that address these interconnected challenges. To address these concerns, healthcare facilities must implement innovative solutions such as resilience training programmes, digital mental health tools, and organisational support systems. One such creative solution is to adapt various AI tools. Novel AI technologies like chatbots, virtual companions, predictive analytics, and AI-driven monitoring systems provide new hope for improving nursing care and the profession (Table 2). For instance, a recent study revealed the development of an artificial intelligence-driven mobile intervention proposing programmes depending on individual needs to minimise nurse burnout. Nurse managers made good use of the tools since they helped to lower turnover and preserve treatment quality. By raising nurse satisfaction, reducing running costs, and improving healthcare institution efficiency, this AI-driven approach raises the quality of healthcare. Personalised AI treatments can, thus, help to lower nurse burnout, increase staff retention, and foster strong and wellbeing-supportive surroundings [8].

## 5. Artificial Intelligence in Nursing and Healthcare

Over the past half-century, AI in healthcare has experienced significant evolution, especially with the introduction of machine learning (ML) and deep learning (DL) technologies [65,66]. In medicine and nursing, these technologies have extended the use of AI and enabled a change toward customised techniques rather than algorithm-only methods [71]. Predictive artificial intelligence models now provide preventative care and disease detection, monitor disease, and streamline clinical workflows to enhance patient outcomes; these models can also increase diagnostic accuracy, expedite clinical procedures, monitor disease and therapy, and improve procedural accuracy [20,72]. The early phases of AI, which occurred between the 1950s and the 1970s, included technologies such as Unimate, the first industrial robot launched in 1961, and Shakey, a mobile robot created at the Stanford Research Institute [73]. These early years focused on the development of devices able to provide typically human-made conclusions based on analysis. Though these developments marked standards in robotics and artificial intelligence, the healthcare sector, especially nursing, fell behind in adopting artificial intelligence since the emphasis was primarily on digitising data necessary for the growth of AI in healthcare. Significant achievements such as the development of early clinical informatics databases and the Medical Literature Analysis and Retrieval System (created in the 1960s) lay a solid basis for the following applications of AI in patient care and nursing support [74]. 

The so-called “AI winter” took place between the 1970s and the 2000s and led to less financing and fewer artificial intelligence discoveries, but notable AI-healthcare partnerships persisted. Clinical researchers were able to network with one another because of the programs developed at the Research Resource on Computers in Biomedicine, Rutgers University (1971), and the Medical Experimental–Artificial Intelligence program, Stanford University (1973) [75]. Early uses included the 1976 CASNET model for glaucoma diagnosis and MYCIN (1972), which examined bacterial infections and suggested antibiotic therapies [76]. The main ways that AI support tools were included in nursing during this period were electronic health records (EHRs) and decision support systems like DXplain (1986), which were developed at the University of Massachusetts [75]. As an electronic medical textbook and a clinical assistant, DXplain helped create differential diagnoses depending on symptoms. DXplain and other EHR systems provided an early layer of artificial intelligence in nursing. In 1980, the robot HelpMate was developed and mainly served as a transport system, but was first installed in hospitals in 1991 to transport materials [77]. While increasing workflow efficiency, these instruments assisted nurses with regular decision-making and patient data management. 

Beginning a transforming phase between 2000 and 2020, new tools and applications were developed to increase the integration of AI in nursing. Paro, a social robot, was created in the early 2000s in Japan and was intended to be a therapeutic companion to patients, especially elderly people [78]. This innovation reduced some of the caregivers’ workload, particularly in long-term care settings. Later, in 2009, Japan developed another robot, Robot for Interactive Body Assistance (RIBA), which could help nurses lift and transfer patients [79]. Designed by Diligent Robotics, Moxi, a nurse-assisting robot, was deployed during the COVID-19 pandemic to handle tasks including cleaning supplies, the delivery and retrieval of medical tools, and specimen handling. Moxi moves independently within hospital settings, reducing nurses’ time on errands, and improves their availability for patient interaction [80].

In 2011, IBM’s Watson represented a significant advancement in AI with its open-domain question answering skills, NLP, and data analysis from unstructured sources [81]. Watson’s applications in medical research, such as the 2017 identification of RNA-binding proteins linked to amyotrophic lateral sclerosis, showcased the promise of AI in evidence-based clinical decision-making [82]. These programs also set the stage for related nursing decision support systems. AI applications in nursing, particularly through EHRs, expanded rapidly during this period by using predictive analytics to track patient outcomes and assist in managing nursing jobs [83]. Chatbots and virtual assistants are being developed using AI to streamline administrative tasks and enhance patient education. Introduced in 2015, Pharmabot gave young patients and their parents medicinal advice [21]. Developed in 2017, Mandy helped simplify patient intake in primary care, reducing the administrative tasks assigned to the nursing staff [84]. DL applications such as convolutional neural networks (CNNs) became extensively used in diagnostic imaging by the late 2010s to enable nurses and other healthcare workers make correct evaluations based on medical pictures [85,86]. These programs replicate neurological processes to allow these experts to make accurate evaluations.

These developments have made AI increasingly critical in nursing since it combines instruments necessary for nursing operations in monitoring, diagnosis, and patient education. More recent uses of AI, such as predictive algorithms to track nurse burnout, can reduce stress and provide customised solutions. These tools show growing dedication to clinical decision-making boosted by AI, efficiency, and patient care quality. AI-driven CDS systems were also designed to analyse real-time data and provide alerts to facilitate early intervention [2,3]. Additionally, AI-integrated automation in administrative tasks, such as scheduling and documentation, can reduce the nursing workload to a greater extent [23,24]. This trajectory emphasises the possibilities of AI to improve nursing, raise job happiness, and lower nurse turnover, helping to establish AI as a fundamental benefit in healthcare and nursing practice.

### 5.1. Chatbots in Nursing

The chatbot, a conversational agent, is an AI-powered computer program designed to simulate human-like conversations. It can chat through text, voice, or visual interactions, allowing users to engage as they would with a natural person [87]. It is one of the critical applications of AI, enabling users to communicate via text-based methods with agents that respond intelligently to their queries. Chatbots can be provided in various forms, such as text-based interfaces or voice assistants. The basic technology used is NLP and DL. NLP lets systems understand and interpret human language, which enables chatbots to perform user queries or commands effectively. Through this technology, chatbots can often include sentimental analysis to gauge user emotions and communicate accordingly [88]. Additionally, large language models (LLMs) have significantly advanced the field of NLP [89]. LLMs are based on neural networks capable of understanding complex linguistic patterns and contextual relations and generating human-like responses.

Due to LLMs, the technology has advanced capability for text recognition, translation, speech recognition, language generation, etc. [90]. These conversational agents can be used to support mental health and are suitable for addressing nurses’ mental challenges. Chatbots like Wysa [69] and Woebot [70] would be best suited for nurses who face stress, burnout, post-traumatic stress disorder, depersonalisation, or any other psychological distress and are also reluctant to seek traditional therapy due to stigma or time constraints. They can deliver cognitive behavioural therapy (CBT), mindfulness, and positive psychology techniques that can reduce stress, anxiety, and depression [70]. A recently published meta-analysis study established the relevance of chatbots in alleviating anxiety and depression [91]. The results highlighted several essential aspects of depression, such as the positive outcome of chatbot-mediated psychological intervention and a short treatment period with efficacy on par with traditional psychological therapy. However, the long-term benefits of chatbots were unclear. Apart from their use in mental health support, they can also help reduce nurses’ workload by automating routine tasks like providing medical reminders, scheduling appointments, and handling casual and repetitive queries from patients [92]. Providing timely reminders and improving patient adherence chatbots can help healthcare providers [93]. Thus, the automation of similar non-urgent tasks can help nurses focus more on direct patient care and reduce their work burden and resultant stress or burnout. However, using chatbots has limitations, such as poor patient adherence due to a lack of consistency or transparency, which genuine human care could provide.

### 5.2. Robots in Nursing

AI-powered robots were first introduced in healthcare in the 1980s [77]. These robots offer a better solution for the growing demands on nurses. They could help nurses reduce their workload by engaging in repetitive and physically demanding tasks, patient monitoring, and clinical decision-making. Robots are a better addition to the healthcare industry in reducing nursing stress and burnout. Though they are not replacements for the human workforce, they are regarded as assistants because they can help nurses and other healthcare providers when the workforce is overloaded or in special conditions like the COVID-19 pandemic [58,94]. They can be deployed in monitoring, testing, pre-diagnosis, delivery of medications and meals, collection and delivery of lab samples, etc. Relay and TUG are examples of robots logistically used in healthcare to carry and deliver medicines, biological samples, etc. [95]. Veebot is a robot that can insert a needle into a patient’s veins to draw blood with an accuracy of 83%. However, the AI-powered advancements in robotics progressed in the early 2010s. ROBEAR, a Japan-made robot, can help patients to stand up or can lift and assist them into wheelchairs. This robot can also recognise faces and voices and respond to voice commands [96]. The robot, Pepper, aided nursing care, especially during the COVID-19 pandemic, in monitoring body temperatures and reminding people to wear masks and disinfect their hands [97]. AI-powered robots use ML, DL, NLP and computer vision, which helps them move around complex environments, avoid obstacles, and interact with patients effectively [4]. While they greatly support healthcare providers, there are concerns about privacy and ethical implications [98].

### 5.3. Predictive Analytics

Owing to the nature of their work, nurses sometimes need to focus on patients who need emergency or critical care; nurses need to prioritise patients, and here, another AI tool—predictive analytics—can assist them. There is a bulk of data in hospitals, such as laboratory results, past medical history, medication history, and allergy information; predictive analytics, which runs on the principle of ML, builds algorithms from these existing data and analyses them to forecast possible outcomes or recommendations [14]. Predictive models can identify risk factors and guide clinical decision-making for better patient care and outcomes. They help healthcare providers tailor unique treatment plans to individual patient requirements and characteristics. The outcomes of such interventions will improve patient satisfaction [99]. From the perspective of healthcare institutions, predictive analytics can be best utilised for efficient resource allocation, maximising efficient healthcare delivery. ML algorithms, such as decision trees, random forests, support vector machines, k-nearest neighbours, and gradient boosting machines, are widely used in healthcare predictive modelling [100].

Some predictive models can analyse and identify high-risk patients regarding disease progression and intervention in chronic diseases like heart diseases, hypertension, and diabetes [1,101,102,103]. For instance, a 2018 study aimed to develop a model for estimating the probability of 30-day readmission in heart failure patients discharged from the hospital. At the optimal categorisation level, the deep, unified network predictive model achieved an accuracy of 76.4% to maximise healthcare cost-effectiveness. Such models help to identify patients with heart failure at high risk of readmission, allowing care teams to focus treatments on those most in need, thus improving clinical outcomes [104]. Similarly, a recent study involved developing and implementing a predictive analytics system to gauge readmission risk in Midwestern hospitals [105]. The study showed that the models effectively guided care teams in timing interventions and adapting strategies through the patient’s further care planning. Another predictive model observed whether a patient could be discharged to a nursing home, allowing hospitals to manage patient capacity and flow for timely surgical care [106].

### 5.4. AI-Driven Patient Monitoring Systems

AI-driven patient monitoring systems let nurses provide more customised and effective treatment by including multiple technologies for different healthcare situations. Remote monitoring systems are especially suitable for monitoring geriatric [107] and chronically ill patients [108] and general and surgical wards [20]. In both inpatient and outpatient environments, wearable gadgets with AI capacity constantly track vital signs like heart rate, oxygen levels, and movement patterns [109]. There are also intelligent beds and in-room sensors in hospitals that help to constantly monitor patients’ vital signs, body position, and mobility [110]. In a study from 2023, patients’ vital signs were monitored using Philips’ IntelligVue Guardian (an automated electronic guidance system), and this approach showed cost-effectiveness and enhanced patient safety [111]. As well as helping nurses with remote patient monitoring, some devices even monitor chronic conditions such as diabetes, hypertension, and heart disease using AI, notifying nurses when readings go beyond safe levels [112]. This remote arrangement helps nurses to intervene as needed and adequately monitor large patient numbers. Especially in intensive care units and postoperative recovery environments, these AI-equipped systems are highly supportive by helping nurses to intervene quickly by identifying early signs of issues such as falls, sleep apnoea, or agitation [110]. Contact-based sensors and wearable devices (Internet of Things (IoT) models) help nurses and other healthcare providers make quick decisions and attend to those in need as soon as possible. With the integration of AI-powered monitoring devices, the strenuous task of manually charting every change in a patient’s condition is now replaced by automatic data logging [113]. These devices reduce the need for hand-held checks, freeing nurses from some of their obligations and freeing time for other, more vital tasks. Overall, these monitoring devices reduce the possibility of significant events and help early response, improving patient safety.

## 6. Benefits of AI to Nurses

### 6.1. AI and Mental Health Support

**Figure 2:**
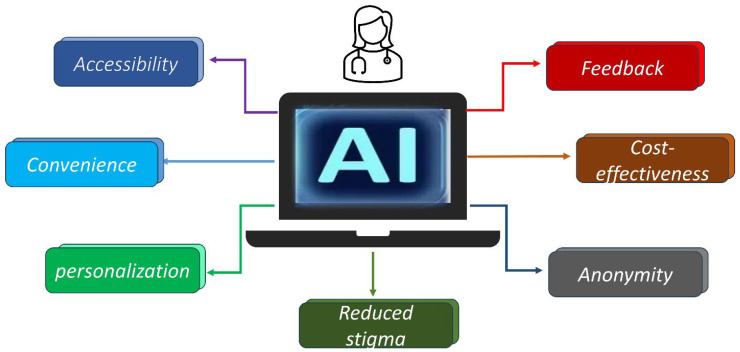
Advantages of AI in mental health support.

The application of AI presents various advantages for the mental distress faced by nurses (Figure 2). While referring to the accessibility of help for nurses in addressing their various mental distresses, the irregular shifts and work schedules are a hindrance. These irregular shifts and work schedules in themselves create physical illness and time constraints, due to which they could have limited access to traditional health services during regular working hours [114,115,116]. With the integration of AI, nurses can manage their work schedules and workloads without compromising patient care. A recent study from Jordan with a group of 112 registered nurses showed that AI supported the nurses in patient monitoring and decision assistance, thereby enhancing time management and work efficiency [117]. While these tools can alleviate nurses’ mental distress, there are also AI-powered mental support systems that they can use at their convenience as they are available 24/7. For instance, Wysa and Woebot offer them real-time support, and these systems provide on-demand mental health support such as mindful exercises, cognitive behavioural therapy, and stress relief exercises [69,70]. It is also noted that mental health stigma remains a significant barrier for nurses seeking traditional support for mental distress [118]. Fear of judgement from colleagues or admitting having mental health troubles can affect their professional reputation. With the support of AI-powered systems, they can maintain anonymity, thereby avoiding these circumstances and, in turn, making the mental health support system approachable [119]. Chatbots like Talkspace and BetterHelp are online treatment systems that link patients with certified therapists using various media, including voice, text, and video chat [120]. Both systems pair patients with therapists who are fit for their particular needs using AI technologies. To meet different mental health needs, BetterHelp offers a more extensive range of therapeutic modalities including cognitive behavioural therapy (CBT) and psychodynamic therapy, whereas Talkspace delivers a simplified experience for linking users to mental health professionals. This type of barrier-free access to mental health support can help to prevent stress and burnout, the two most prevalent issues.

Another benefit of using AI systems is that they provide personalised mental health support, addressing the unique needs of individual nurses [121]. Using inputs and behavioural patterns, AI algorithms can generate tailored recommendations, thus increasing the quality of personalised, accurate feedback. Shine is an example of an AI tool that provides tailored daily motivation and assistance. It uses AI to identify users’ needs and preferences, providing tailored materials and resources for them [120]. Interventional tools should be able to adapt dynamically to the evolving needs of the users. Using gamification ideas and affordance-based design, gamified smartphone software for mental health management tool was developed that lets users participate in deep breathing exercises [122]. The device uses ML to provide real-time biofeedback, guiding users through breathing exercises with interactive visuals and modifying to fit individual breathing patterns to generate customised recommendations. The results showed notable declines in stress markers and higher user engagement, highlighting the possibilities of AI-driven therapies to enhance mental health outcomes.

Other AI tools like Ginger and Woebot continuously monitor users’ interactions and adjust their interventions accordingly [123]. Nurses who use them receive real-time feedback, which helps them to manage stress before it escalates. Apart from real-time feedback, these systems can also analyse biometric data from wearables and smartphones. These data could be obtained from sleep patterns, heart rate, or activity levels. Such levels of personalisation enhance the overall result of interventions and encourage sustained engagement with the mental health support system with no additional cost, unlike traditional therapies.

AI-powered mental health support systems present much more cost-effective alternatives, and some platforms are free or at a minimal subscription fee compared to traditional therapy [69]. The cost-effectiveness would be feasible for healthcare institutions and individual nurses, who might struggle to afford regular therapy sessions. A study in 2017 highlights this aspect of AI-based mental health interventions, especially in large healthcare systems where nurse burnout is prevalent [124]. In another study involving 75 individuals from 15 different US colleges, an AI-driven mental health tool called Tess underwent a randomised controlled experiment [125]. The trial’s goal was to ascertain whether Tess helped to reduce depression and anxiety symptoms. Three groups were formed from the participants: two groups had free access to Tess, one group had daily check-ins for two weeks, and the other group had bimonthly check-ins for four weeks. Conversely, the control group consisted of just receiving an instructional link to the eBook of the National Institute of Mental Health. The results showed that Tess significantly helped both intervention groups to lower their anxiety and depression symptoms when compared to the control group. This suggests that Tess influences mental health very well. Furthermore, members of the intervention groups claimed better mood, which emphasises Tess’s possibilities as a reasonably affordable and readily available support aid for the treatment of mental health issues. Tess is not meant to be a trained therapist. Still, AI technologies like Tess can be used as scalable and reasonably priced options for providing psychological help.

### 6.2. AI Support in Nursing Care

Using advanced technology, AI-integrated nursing care helps nurses to deliver more efficient treatment and optimises workflows and patient outcomes. Combining AI-driven solutions with traditional nursing practices helps hospitals to satisfy complex patient needs and reduce nursing staff workloads [13]. Automating routine activities such as vital sign monitoring and data collection helps create effective workflow and time management by freeing nurses to focus on direct patient care [108]. Besides monitoring, automated alarms and notifications for critical test results or changes in patient conditions improve emergency response capacity [121]. Thus, real-time data and clinical decision assistance, which are readily available and support evidence-based treatment and educated clinical judgement, can improve therapeutic results. Concerning work schedules, when automated, it can reduce disputes and guarantee a sufficient workforce, improving the efficiency of administrative chores [117]. Better nursing care could be offered to patients if nurses endure less documentation and can incorporate AI tools into specific jobs to improve accuracy and optimise billing and documentation [25]. Using EHRs ensures complete, current patient records, reducing errors connected to manual record-keeping and improving patient care and safety [65]. Moreover, pharmaceutical management methods using barcoding and automation greatly reduce drug mistakes, enhancing patient safety [126].

Communication between nurses, patients, and other healthcare staff must also ensure safe and quality nursing care. Effective communication influences patient education, adherence to the treatment guidelines, early identification of health issues, and general patient satisfaction [127]. By giving patients access to health education resources, technology helps improve their understanding of their diseases and treatments, enhancing patient education and involvement. Wearable remote monitoring helps patients track their health, encouraging self-care and increased participation in health management [22]. AI applications like telehealth and telemedicine allow doctors and nurses to offer care to patients from afar, especially in underdeveloped areas. Digital tools also make it easy for healthcare workers to work together, which improves care coordination [18]. Even chronic diseases could be managed by AI. For instance, DIABTel is a telemedicine system used to manage patients with type 1 diabetes [19]. The system consists of a clinical operating system used by hospital nurses and doctors and a patient unit used for daily activities by patients. It is possible to collect, manage, and interpret data and allows the exchange of data and messages, thereby ensuring prompt care.

## 7. Challenges in Implementing AI in Nursing

Though there are sufficient reasons to suggest that AI could enhance nursing care, significant challenges exist in the healthcare context. Some of the challenges are mentioned below.

### 7.1. Reliability and Accuracy

AI systems applied in health assessments usually depend on algorithms that generate false positives or negatives, which can affect diagnosis and treatment [128]. Owing to this, concerns have been raised about the reliability and accuracy of AI systems. AI sometimes shows algorithmic biases, aggravating this issue considerably since they could disproportionately impact marginalised populations [129]. AI models’ training datasets may not entirely reflect the patient populations they are meant to serve. An example of this issue is given by a 2019 study by Obermeyer et al. Their analyses revealed that AI algorithms applied to predict health risks often overstated the care demands of black patients in contrast to white patients, therefore generating uneven treatment [130].

Avula et al. looked at the quality of data provided by AI tools in the healthcare sector and found incompatibilities in some AI systems [131]. Any flaw in analysis or recommendation can increase the chance of misinterpretation due to missing or outdated information. The possibility of errors in decision-making and treatment outcomes because of AI support in healthcare is also mentioned elsewhere [132]. Unreliable or inaccurate results could be produced due to data merging [133]. Many hospitals and clinics use several software tools, and these technologies may not always work together. There is a lack of standardisation of these AI solutions in the healthcare sector, developed independently by several companies. Among the several data sources that nurses are expected to handle, some of the most often used ones include patient reports, wearable technologies, and EHRs. Organising all of these distinct data in a way that AI systems can understand and exploit can be difficult. For example, AI-driven systems that help nurses monitor patients can spot problems, but these systems might not always have expert judgement from years of work experience [134].

### 7.2. Data Privacy and Security

The healthcare sector accounted for 15% of all global data breaches in 2017, the second-highest percentage at that time [135]. Data privacy and security are essential for applying AI in nursing, especially for delicate patient data like mental health records [136]. Exceptionally delicate information about mental health may be used or exposed to cyberattacks if it is not securely guarded [137]. The spread of AI technologies has heightened these concerns in the healthcare sector. For instance, in 2015 alone, 112 million healthcare data records were breached [138]. Since AI systems often use cloud-based servers to handle data, they make it easier for hackers to obtain access to systems. Many AI models are trained on massive datasets, including personal information, so it is crucial to safeguard these data both during storage and transmission [139]. Apart from the risks caused by outside players, internal stakeholders have voiced concerns about data abuse [140]. Patients may suffer greatly regarding reputation from the exposure or use of healthcare data, including discrimination, stigmatising, insurance loss, or unemployment [141]. Given these issues, strict government and privacy standards must be followed. For instance, regulatory bodies like the General Data Protection Regulation (GDPR) in the European Union and the Health Insurance Portability and Accountability Act (HIPAA) in the United States came into existence to help reduce these risks [142].

Inaccurate assessments compromise the quality of the given treatment and confidence in the technology. Regarding mental health assessments, nurses who depend on AI systems could be obliged to make tough ethical decisions should the AI recommendations contradict their clinical judgement. In a recent study on AI-assisted decision-making in nursing practice, the participants pointed out that depending too much on the AI system could lead to misguidance [143]. Further, they stated that this could lead to inappropriate care strategies and negative effects on nursing care resulting from unforeseen events or erroneous or suboptimal recommendations by the system. Being a technological assistant in healthcare lacks human qualities, unlike nurses, who lack contextual awareness. Hence, it may misinterpret circumstances owing to the speed with which information is processed [144].

### 7.3. Resistance to Adoption

Nurses have hesitated to incorporate AI in clinical applications as they mostly do not understand the process or logic it uses [145]. A recent study from Saudi Arabia found that nurses had only a moderate level of knowledge about AI, and only slightly over half (58.2%) of the participants had any practical knowledge of AI-integrated technology [146]. This study’s analysis also showed a conservative attitude towards AI, which extends from mild caution to considerable resistance against incorporating AI into nursing practice. This apprehension arises from fears of job replacement, mistrust towards AI regarding reliability and accuracy, other ethical concerns about job displacement, doubts regarding AI’s reliability and accuracy, and ethical fears concerning patient privacy and inadequate training. Because of their complex algorithms, AI models, commonly called black boxes, make it difficult for health practitioners to understand how they conclude decision-making or suggestions [147]. Particularly in high-stress settings like intensive care units, it is not unusual for nurses to voice concerns about the validity of AI solutions [148,149]. Hence, there is a lack of trust in AI systems in critical situations and resistance to adoption into healthcare. A study from 2023 found that 23% of nurses feared AI, and 36% had some negative attitude towards using AI [150]. Researchers recommend including AI awareness and training in nursing education, sprouting from the results of their study that 74.8% of nurses lacked a solid understanding of AI, and 12.7% of the participating nurses perceived AI as a threat to them rather than an opportunity [151]. Consistent with this study, another group of nurses showed that 70% had inadequate or no knowledge about AI techniques [152]. Most of the literature points to the fact that there is resistance to AI adoption primarily due to a lack of awareness and knowledge that could prevent nurses from effectively utilising the possibilities of AI and, hence, not exploiting the application to its full utility or even making mistakes while using it [153].

## 8. Ethical Implications of AI in Nursing 

**Figure 3:**
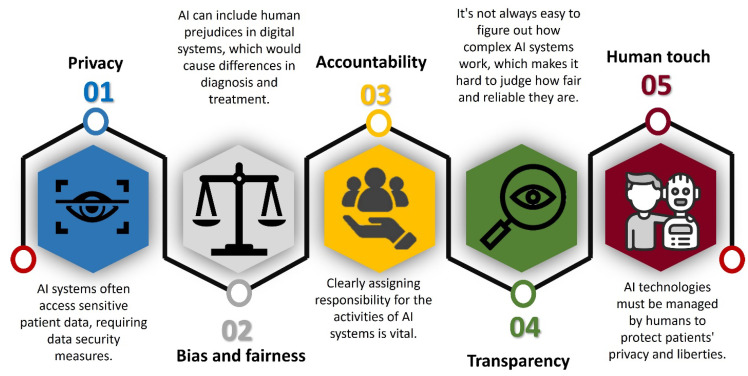
Ethical implications of AI in healthcare.

AI integration in nursing also presents some critical ethical concerns (Figure 3), such as privacy, bias, and accountability, along with the necessity to preserve the fundamental “human” aspect of nursing [154]. Examining these ethical issues will help guarantee the correct use of AI capacity while safeguarding patient welfare and the rights of both patients and nurses.

AI typically depends on large databases, including personal data, to improve treatment quality. Sensitive data, particularly from monitoring devices that could record physiological and psychological metrics including emotional states, are prone to privacy issues [137]. Even though these data-driven insights have many clear advantages such as the early identification of health hazards, they also pose the danger of exposing private data as these platforms are prone to breaches. The data utilised in AI algorithms can be shared with third-party entities without intending to do so [155]. This could result in the prospect of exploitation, therefore undermining patient faith in systems like AI. The privacy of nurses is also in danger, especially in hospitals where AI algorithms assess staff performance and workload and employee data in hospital systems [156]. These systems may collect and analyse sensitive employee information without explicit consent or adequate anonymisation, raising privacy concerns. In the event of a cyberattack, the whole hospital system could come under threat, including the leak of personal information of patients and staff [157]. A pronounced example of this is the 2017 “WannaCry” ransomware attack affecting 230,000 computers in 150 countries and disrupting the operations of the National Health Service (NHS) in England [158]. In this incident, hospital staff were locked out of their computers, and hackers demanded payment to restore access to encrypted data [159]. This incident stressed the susceptibility of healthcare systems to ransomware attacks, with far-reaching impacts on patient care and hospital operations.

All health sectors are concerned about data privacy and the risk of AI systems mismanaging personal information [141]. To handle privacy concerns effectively, robust security policies and open data governance must be followed. This will guarantee that data concerning nurses and patients are preserved safely and used for authorised purposes. 

Another ethical concern with AI technology is AI bias, often leading to inaccurate or suboptimal treatment outcomes [160]. AI biases can lead to serious consequences by reinforcing societal biases. When specific groups, such as gender and ethnic minorities, are underrepresented in big data, AI systems may misdiagnose these patients more often with undesirable healthcare outcomes [161]. For instance, a bias in the algorithm assessing eligibility for kidney transplants favouring certain demographic groups resulted in a delay in kidney transplants amongst the minority group [162]. Thus, AI models repeatedly produce erroneous findings due to variations in how demographic variables such as gender, colour, or age are expressed in datasets. Another study showed how exclusion led to the underrepresentation of African Americans in clinical studies [163]. To solve such concerns, when designing AI tools, the developers should be able to combine diversity into algorithm training datasets, fairness-oriented data mining techniques, and frequent evaluations to identify and correct discriminating results. One of the ways health systems can lower bias is by assembling diverse teams comprising professionals from clinical, social, and technical domains. These groups are more suited to identifying possible prejudices and create fair and inclusive activities. Moreover, including a spectrum of patients and local populations in developing clinical algorithms enables us to consider various requirements and points of view. This can lead to framing algorithms that better aid underrepresented groups [164].

The next important ethical concern is accountability and transparency [154]. Complex models such as neural networks can provide highly accurate forecasts without information on how judgements are made, which makes it challenging for healthcare providers to depend entirely on these outputs since they typically act as “black boxes” [147]. Transparent and ethical AI systems enable the avoidance of possibly negative or permanent consequences on patients, promoting responsible and reliable AI utilisation for important medical purposes [165]. AI models that clearly explain their decisions can achieve transparency. They also need clear lines of accountability for choices affecting AI-driven systems’ results. This would help nurses have better confidence in their work and enable them to hold those in charge of mistakes liable should they happen [166]. Several methods can achieve transparency: explainable AI, open-sourced algorithms, transparent data practices, and regular audits [154]. However, enabling transparency has several challenges, including implementation complexity, security risks, and resource-intensive maintenance.

One of the most important and core ethical considerations is balancing technology and the “human touch”. The nursing profession embodies profound significance through the essential values of human touch and compassionate care [167]. This is because the “human touch” determines the mental state of nurses and patients. In mental healthcare, where human interaction can be equally crucial as treatment, the sympathetic bond between nurses and patients usually results in therapeutic gains. A recent study reported human touch in care as a challenge in AI–healthcare integration [168]. Yet another study emphasises that regardless of technological convenience, AI integration should be approached carefully due to the significance of the nurse–patient relationship [108], thus stressing the need to find a balance between the efficiency of technology and the provision of personal care. This would demand creating a setting where empathy and technology coexist to raise the standard of treatment without erasing the nurse–patient relationship. Appropriate attention to definite policies, transparent algorithms, and fair procedures could assure ethical integration and accountability. Addressing these concerns will make tremendous progress in AI-integrated nursing care.

## 9. Future Directions and Recommendations for AI in Nursing

Studies have corroborated that AI transforms nursing practice by increasing productivity, lowering stress, and improving patient care quality. For instance, in a study on intelligent surveillance systems, AI was demonstrated to reduce nurses’ 18 min- to 10 min interaction time with patients [169]. Real-time updates on the patient’s health situation helped them to do this. This helped nurses to focus on delivering vital treatment and avoid subsequent injuries and patient crises. A study conducted in Colombia revealed that more than half of nurses’ time is spent on non-professional activities [170]. These tasks include waiting for the doctor’s approval (which may take 85 min daily) and inputting duplicate data, which might be automated to save up to 10% of their time and maximise resource utilisation. Based on studies on robot-assisted nursing, 67.2% of nurse managers said that, without replacing nurses, robots might significantly lower workloads [171]. Integrated studies also underlined the value of robots in delivering medications and patient monitoring, enhancing safety and satisfaction. Moreover, there is a relationship between the use of robots to help with professional work and increased job satisfaction, as well as claimed improvements in health, which, in turn, lowers the possibility of nurses leaving their present jobs. Research comprising 331 nurses carried out in Taiwan confirmed these benefits [172]. The study underlined how robotic technologies could help nurses focus their efforts from non-professional tasks into professional ones. Furthermore, another study revealed that AI technologies, such as CDS systems, have helped simplify processes. Forty per cent of nurses have reported better patient monitoring, thirty-six per cent have experienced reduced stress levels, and twenty-seven per cent have noted more accuracy in daily tasks whilst using AI [173]. The accomplishment of an 87% agreement rate with the diagnosis given by nurses by means of adaptive-network-based fuzzy inference systems is another illustration of the superior diagnostic accuracy demonstrated by AI-powered nursing information systems [174]. Apart from cutting the non-essential time spent working, these technologies improved the quality of data collection, thereby enabling faster response to issues related to patient health. It has been shown that humanoid robots, such as Pepper, could increase interaction with the long-term care of dementia patients [171]. However, to fulfil their potential, more technical developments are needed. Together, these results show the transforming power of robotics and AI in lowering workloads, improving diagnosis accuracy, and supporting nurse wellbeing while concurrently sustaining high-quality patient care.

However, ongoing research is essential to validate the use of AI technologies in nursing. This guarantees that these technologies are based on facts and tailored to satisfy nursing care requirements. AI will likely use general solutions that overlook nurses’ specific circumstances, such as high-stress environments and patient diversity, in the lack of validation. Studies conducted in the field of AI support this [175,176]. Developers can establish their safety and efficacy through trials and longitudinal studies on AI products. Using their respective applications, these technologies are meant to lower nursing burnout risk, increase patient care accuracy, and boost mental health. Customised AI systems must include input from nurses and stress practical applications, including patient triage, mental health surveillance, and task management, to serve nursing professionals’ objectives properly.

Nursing education has to include AI if we want to raise healthcare workers’ degree of understanding and readiness concerning this technology. Incorporating AI training into nursing courses will help to ensure that future nurses fully grasp the possibilities and constraints of AI within the framework of the healthcare sector. This might promote early acceptance of AI and help to lower the usually adverse reaction against technological advancement. If nurses had primary training in algorithmic decision-making or patient data analysis, they would be more suited to working successfully with AI tools. AI methods would help them enhance clinical judgement instead of replacing it, benefiting society. Early involvement with AI can help produce a workforce confident in its capacity to employ AI technology responsibly in medical care settings.

Clear policies and ethical standards help to guarantee that AI is applied with responsibility in nursing. Since healthcare is a sensitive topic, these guidelines should concentrate on ensuring that AI helps to maintain patient and staff safety. Emphasising that privacy and morality are the most critical concerns in these kinds of AI uses, laws could demand explicit algorithms, safe data, and moral usage in mental health tracking. Policies supporting “explainable AI” ensure that nurses comprehend and accept the recommendations of the AI, therefore ensuring that its decisions correspond with the nursing code of ethics and values stressing the patient.

Working together, technologists, medical professionals, and legislators should produce safe and morally and practically effective AI solutions. By working with healthcare professionals, AI developers can create solutions that satisfy practical needs by integrating modern technologies with the experience of daily caregivers. Although legislators support the proper use of AI, they also significantly contribute to creating legislation safeguarding the interests of providers and patients. By working collaboratively, nurses can employ AI to enhance treatment; one way to accomplish administrative tasks to lower staffing requirements is by automation. This can be achieved while acknowledging the vital human and personal nursing elements.

## 10. Conclusions

AI has shown great promise in transforming how nurses obtain mental health support by developing fresh approaches to handling complex issues like burnout, stress, and compassion fatigue. AI tools like predictive analytics, virtual companions, and tracking systems run by AI help mental healthcare and help nurses and technology collaborate more efficiently. The capacity of AI to forecast the future allows healthcare systems to identify which nurses are more likely to have mental health issues, enabling quick assistance for them and lowering occupational stress. People can also use virtual companions and chatbots, making mental health resources available twenty-four hours a day, seven days a week. These instruments offer individualised help in addition to conventional treatment. These advancements demonstrate how well AI can help protect nurses’ emotional and mental wellbeing, enhancing patient care and easing their employment. Nonetheless, using AI in nursing care must have a clear ethical foundation. Protecting data privacy, addressing biases, and maintaining open AI systems help avoid ethical issues and foster trust in healthcare environments. Any AI solution designed to support nurses’ mental health must abide by rigorous data security policies if we are to keep this information safe. While maintaining the compassionate, person-centred approach at the core of nursing, one should also consider how AI tools might alter how a nurse and patient engage. AI should enhance rather than replace the loving ties that define nursing care. We must ensure that AI is thoroughly used in nursing in the future, using additional research, wise investments, and monitoring of ethical issues. Legislators, research institutes, and hospitals should cooperate to create comprehensive guidelines supporting morally sound and effectively operating AI capabilities. Training courses should also be designed to inform nurses about the advantages and drawbacks of AI so that they may apply these tools with assurance and effectiveness. Using AI wisely would enable the healthcare sector to strengthen its staff, enhance patient care, and safeguard nurses’ health.

## Figures and Tables

**Table 1:** Benefits of AI in nursing.

Benefit	Description	Reference
Improved Patient Care and Safety	- Accurate Documentation: EHRs ensure comprehensive, up-to-date patient records, minimising errors in manual records.	[17]
	- Medication Management: Barcoding and automated systems reduce medication errors.	
Enhanced Communication and Collaboration	- Telehealth and Telemedicine: Enables remote consultations, monitoring, and care for patients in underserved areas.	[18,19]
	- Care Coordination: Digital tools enable seamless collaboration among healthcare teams.	
Efficient Workflow and Time Management	- Automation of Routine Tasks: Vital sign monitoring and data collection automation, freeing up time for patient care.	[20]
	- Quick support: Quick access to guidelines and patient information aids rapid decision-making.	
Real-Time Data and Clinical Decision Support (CDS)	- Evidence-Based Care: Access to current research and guidelines supports informed clinical decisions.	[2]
	- Alerts and Notifications: Automated alerts for critical labs or condition changes enhance emergency responses.	
Enhanced Patient Education and Engagement	- Health Education Resources: Technology provides educational materials to improve patient understanding.	[21,22]
	- Remote Monitoring: Wearables allow patients to track their health, enhancing self-care and engagement.	
Increased Efficiency in Administrative Tasks	- Scheduling and Staffing: Automated scheduling ensures adequate staffing and reduces conflicts.	[23,24,25]
	- Billing and Documentation: Streamlined billing and documentation reduce paperwork and improve accuracy.	

**Table 2:** Overview of AI integration in nursing.

Type of AI	Description	Example of Healthcare Application	Reference
Predictive Analytics	Forecasts patient deterioration, enabling early intervention	Early warning systems (e.g., heart rate observation that alert nurses about potential patient risks	[64]
Natural Language Processing (NLP)	Assists in clinical documentation and extracts critical information from patient records	DeepCura, Nightingale Notes by Trusted Health, Suki AI Assistant for generating nursing care plans based on patient records	[65]
Machine Learning (ML)	Analyses data to classify and predict patient conditions	Sepsis Watch model to predict sepsis onset, supporting faster nursing response	[66]
Robotic Process Automation (RPA)	Automates administrative tasks, reducing workload	Automating appointment scheduling and medication reminders for patients	[67]
Computer Vision (CV)	Interprets visual data for patient monitoring and alerting nurses	Physiologic monitoring or detection of patient movement	[68]
Conversational AI	Engages in patient communication and symptom assessment	Chatbots like Wysa and Woebot provide mental health support to reduce nurse workload	[69,70]

## Data Availability

All data related to this study are provided in the manuscript. The data presented in this study are available on request from the corresponding author.
